# New insight into the structures and formation of anthocyanic vacuolar inclusions in flower petals

**DOI:** 10.1186/1471-2229-6-29

**Published:** 2006-12-17

**Authors:** Huaibi Zhang, Lei Wang, Simon Deroles, Raymond Bennett, Kevin Davies

**Affiliations:** 1 New Zealand Institute for Crop & Food Research Limited, Private Bag 11-600, Palmerston North 4442, New Zealand; 2 Previous address: The Horticulture and Food Research Institute of New Zealand Ltd, Private Bag 11 030, Palmerston North 4442, New Zealand

## Abstract

**Background:**

Although the biosynthetic pathways for anthocyanins and their regulation have been well studied, the mechanism of anthocyanin accumulation in the cell is still poorly understood. Different models have been proposed to explain the transport of anthocyanins from biosynthetic sites to the central vacuole, but cellular and subcellular information is still lacking for reconciliation of different lines of evidence in various anthocyanin sequestration studies. Here, we used light and electron microscopy to investigate the structures and the formation of anthocyanic vacuolar inclusions (AVIs) in lisianthus (*Eustoma grandiflorum*) petals.

**Results:**

AVIs in the epidermal cells of different regions of the petal were investigated. Three different forms of AVIs were observed: vesicle-like, rod-like and irregular shaped. In all cases, EM examinations showed no membrane encompassing the AVI. Instead, the AVI itself consisted of membranous and thread structures throughout. Light and EM microscopy analyses demonstrated that anthocyanins accumulated as vesicle-like bodies in the cytoplasm, which themselves were contained in prevacuolar compartments (PVCs). The vesicle-like bodies seemed to be transported into the central vacuole through the merging of the PVCs and the central vacuole in the epidermal cells. These anthocyanin-containing vesicle-like bodies were subsequently ruptured to form threads in the vacuole. The ultimate irregular AVIs in the cells possessed a very condensed inner and relatively loose outer structure.

**Conclusion:**

Our results strongly suggest the existence of mass transport for anthocyanins from biosynthetic sites in the cytoplasm to the central vacuole. Anthocyanin-containing PVCs are important intracellular vesicles during the anthocyanin sequestration to the central vacuole and these specific PVCs are likely derived directly from endoplasmic reticulum (ER) in a similar manner to the transport vesicles of vacuolar storage proteins. The membrane-like and thread structures of AVIs point to the involvement of intravacuolar membranes and/or anthocyanin intermolecular association in the central vacuole.

## Background

Anthocyanins are a large subclass of flavonoid pigments [1] that provide important functions in plants and are also of significance to agriculture and commerce [2]. Their biosynthetic pathway, a branch of phenylpropanoid biosynthesis, has been extensively characterized, and there is also a good understanding of the transcriptional regulation of the structural enzyme genes [3-5]. Furthermore, they are one of the few groups of secondary metabolites for which there are data on the sub-cellular nature of the biosynthetic enzyme complex and the subsequent transport of the phytochemical product to the site of accumulation. Anthocyanins are synthesized in the cytoplasm, likely by a multienzyme complex anchored on endoplasmic reticulum (ER) via the cytochrome P450 enzymes that are part of the complex [6,7]. Once formed, the anthocyanins are transported from the cytoplasm into the vacuole, an acidic environment in which anthocyanins can accumulate to high levels, and in which they assume a brightly colored chemical structure [7]. Although there has been progress from molecular studies in deciphering the molecular requirement of the transport process to the vacuole, this is the least understood stage of the biosynthetic pathway at cellular and sub-cellular levels.

There is evidence from several species for a number of alternative transport routes relating to intracellular transport of the flavonoids, with anthocyanins being possible targets for only some of these. Some members of the glutathione S-transferase (GST) family have been found to be necessary for anthocyanin sequestration into the vacuole [8-11]. Although a mechanism similar to xenobiotic detoxification processes was proposed for anthocyanins [8], specifically addition of glutathione residues by GST to form stable water-soluble conjugates and the sequestration of these conjugates by ATP-binding cassette (ABC) transmembrane transporters, no anthocyanin-glutathione conjugates have been observed *in vivo*. Instead, the GST works as an anthocyanin-binding protein that may escort anthocyanins from the synthetic site to the tonoplast [12]. A second possible transport route is via multidrug and toxic compound extrusion (MATE) transporters located in the tonoplast membrane. Mutant analysis has suggested the involvement of a MATE transporter for proanthocyanins in Arabidopsis [13], anthocyanins in tomato (*Solanum lycopersicum*, [14]) and maize (*Zea mays*, [15]).

A third aspect of proanthocyanin/anthocyanin transport is the coordination of the transport process with vacuole biogenesis, and the involvement of vesicles. Black Mexican Sweet (BMS) suspension cell lines of maize transformed with maize anthocyanin transcription factor transgenes produce high levels of phytochemicals, and also trigger the production of autofluorescent vesicles that are transported into vacuoles [16,17]. Furthermore, the *tds4 *mutation of Arabidopsis, that prevents anthocyanidin synthase activity and inhibits proanthocyanin production, prevents normal vacuole development and causes accumulation of small vesicles [18]. This effect is not seen with mutations affecting other enzymes in the proanthocyanin biosynthetic pathway, implying a link between proanthocyanin biosynthesis and vacuole development. Thus, one possibility is that the major vacuole in a pigmented cell may grow by small anthocyanin-containing pro-vacuolar vesicles being formed at the site of anthocyanin biosynthesis, which then bud off the ER and fuse with the tonoplast.

With regard to the fate of the anthocyanins after transport to the central vacuole, a number of different forms of anthocyanin accumulation have been observed with light microscopy: an evenly colored solution, vesicle-like bodies and dense, compact bodies of either regular or irregular shape. Some of the anthocyanin-concentrated bodies in cells were originally suggested as sites of anthocyanin biosynthesis, and termed anthocyanoplasts [19]. However, upon their further characterization they have been given the name Anthocyanic Vacuolar Inclusions (AVIs) [20]. AVIs have been found in a wide range of angiosperm species [19], without any obvious phylogenetic association. The most studied vesicle-like AVIs are those observed in suspension cell cultures of sweet potato (*Ipomoea batatas*) and maize. In sweet potato, the vesicle-like AVIs usually start as a large number of smaller vesicles that gradually fuse into a small number of larger vesicles [21]. No boundary membrane has been observed for these sweet potato AVIs [22]. However, specific proteins have been found associated with the AVIs [22].

AVIs in petals of lisianthus (*Eustoma grandiflorum*) and carnation (*Dianthus caryophyllus*) have been reported to be non-vesicle, dense and compact bodies, which can be isolated from the plant as particles [20]. It was reported that the AVIs of lisianthus do not have a surrounding membrane, but, as with sweet potato, may have protein components that are involved in selectively binding specific anthocyanin structures. The association of anthocyanins with AVIs in lisianthus is also thought to shift the perceived petal color [20].

In this study, we report on the further characterization of the AVIs of lisianthus. Using a combination of light microscopy, TEM and SEM the structural aspects of AVIs *in planta *and as isolated particles have been studied, and evidence obtained for their formation from ER-derived vesicles.

## Results

### Forms of AVIs in different petal regions

**Figure 1:**
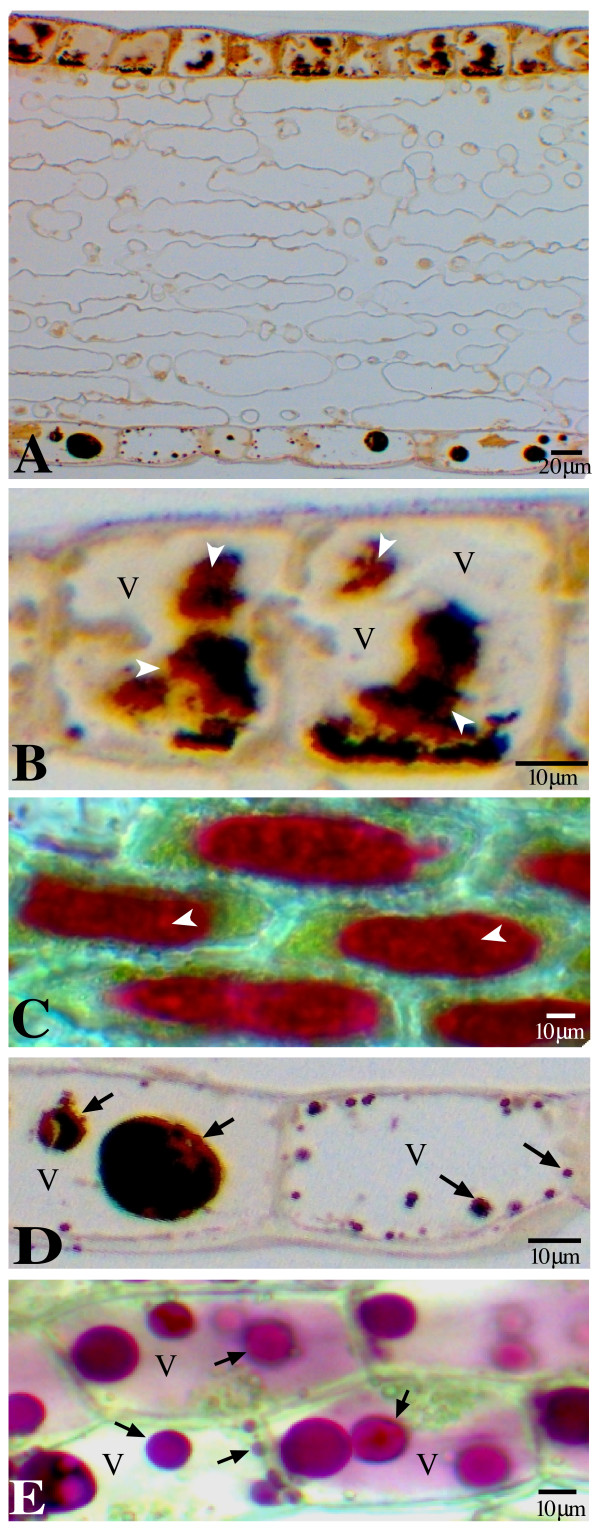
**Micrographs of AVIs in the epidermal cells of fully open lisianthus petals**. A. Bright field microscopy image of an unstained transverse section of the inner petal region, showing the distinct morphology of AVIs between the adaxial and abaxial epidermal cells. Irregular AVIs in the adaxial epidermal cells (upper) and vesicle-like AVIs in the abaxial epidermal cells (lower). B. Transverse section of adaxial epidermal cells in Fig. A at higher magnification, showing the central vacuoles (V) and the irregular AVIs (arrowhead). C. Adaxial epidermal peel of the inner petal region under bright field, showing the irregular form of the red-colored AVIs (arrowhead). D. Transverse section of abaxial epidermis of the same inner petal region, showing vesicle-like AVIs (arrow) and central vacuoles (V). E. Abaxial epidermal peel of the inner petal region observed under bright light, showing vesicle-like AVIs (arrow) and central vacuoles.

The petals of lisianthus have a dark inner throat region and a lighter colored outer region, with anthocyanins present in both the abaxial and adaxial epidermal cells. The shape difference of AVIs between the outer and inner petal regions of lisianthus flowers was noted by previous researchers [20]. In this study, we investigated the form variations of AVIs in the epidermal cells located at different regions of lisianthus petals. Microscopic examination of the unstained transverse sections under bright field showed that not only are the AVI forms in the adaxial epidermis different between the outer and inner petal regions, but also the AVI forms differ more greatly in the adaxial epidermal cells than in the abaxial epidermal cells of the same inner petal region (Fig. 1A). The dark brownish color that remained in the epidermal cells of the transverse sections provided a good marker to recognize the anthocyanin-containing structures.

In the adaxial epidermis (Fig. 1A and 1B), the brownish AVIs in the central vacuoles displayed irregular forms, sizes and even appeared as separated masses in transverse sections. These AVI structures did not appear to be highly organized and seemed to have loose 'fuzzy' structures (Fig. 1B), with the main AVI body occurring towards the centre of the main vacuoles. Around this loose structure, a highly colored band was apparent in most AVI-containing adaxial epidermal cells (Fig. 1A). The AVIs in the freshly peeled adaxial epidermis (Fig. 1C) of the inner petal showed intensely colored AVIs in the main vacuoles and very uneven surfaces of these AVIs were observed from the top under a bright field microscope (Fig. 1C), again displaying loose structures of the anthocyanin-containing deposits.

**Figure 2:**
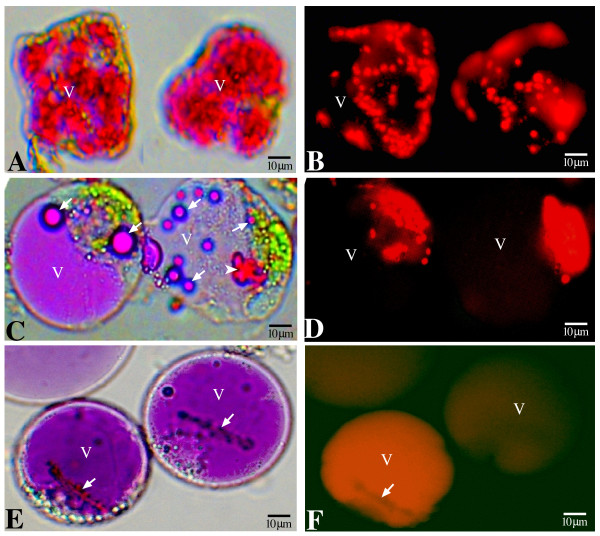
**Morphology of AVIs in isolated protoplasts derived from the different epidermal cells. V, central vacuole**. A. Bright field microscopy image of protoplasts isolated from the adaxial epidermis of inner petal region, showing rigid shape of the protoplasts and AVI consisting of granules and threads. B. Fluorescent microscopy image of the same protoplasts shown in A. Red color showing chloroplasts emitting red auto-fluorescence from chlorophylls. C. Bright field microscopy image of protoplasts isolated from the abaxial epidermis of the inner petal region, showing vesicle-like AVIs (arrow) in the round protoplasts. Chloroplasts, green. D. Fluorescent microscopy image of the same protoplasts shown in C. Chloroplasts revealed by the red auto-fluorescence. E. Bright field microscopy image of protoplasts isolated from the adaxial epidermis of the outer petal region, showing the presence of rod-like AVIs (arrow). F. Fluorescent microscopy image of the same protoplasts shown in E. No chloroplasts revealed.

Protoplasts generated from adaxial epidermal cells of inner petals displayed more dispersed AVIs than were apparent in the epidermal peel (Fig. 2A), clearly showing irregular-shaped anthocyanin-containing deposits of the AVI. Interestingly, these AVI-containing protoplasts were rigid, tending to maintain their original cell shape. No cell walls were evident under fluorescent microscopic examination of these rigid protoplasts, as cell walls would have given clear cellular borders under the UV lighting condition used (Fig. 2B). Furthermore, the protoplasts generated from the adaxial epidermis of the inner petal region also contained functional chloroplasts as revealed by red auto-fluorescence emitted from chlorophyll (Fig. 2B).

AVIs in the abaxial epidermal cells of the inner petal region were vesicle-like bodies of varying sizes (Fig. 1D and 1E). The protoplasts generated from these abaxial epidermal cells were round with a large colored vacuole and vesicle-like AVIs scattered in the vacuole and cytoplasm (Fig. 2C). Occasionally, anthocyanin-containing deposits similar to those observed in the adaxial cells were also seen in these abaxial protoplasts (Fig. 2C). Chloroplasts were also present in these abaxial protoplasts as shown by the red autofluorescence (Fig. 2D).

The protoplasts generated from adaxial cells of the outer petal region had a round shape with a large colored vacuole (Fig. 2E). Single barbed rod-like AVIs were present in each of the highly colored vacuoles of the protoplast (Fig. 2E). No chloroplasts were observed in these protoplasts (Fig. 2F).

### Topographic features of AVIs

**Figure 3:**
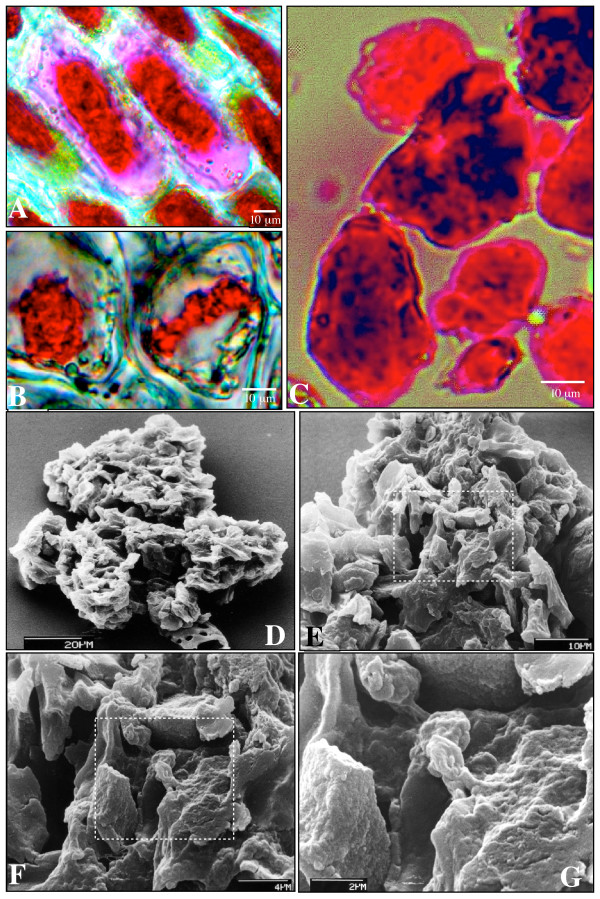
**Topographic micrographs of lisianthus AVIs**. A. Bright field image of AVIs in adaxial epidermal cells of the inner petal region, showing the surface structures of the AVIs (red) and the weakly colored area (pink) surrounding these AVIs. B. Bright field image of AVIs in the central vacuoles of the adaxial epidermal cellsof the outer petal region. C. Bright field image of isolated AVIs mounted on glass slide in 0.1 M PBS (pH 7.0), AVIs showing colored threads and granules. D. SEM image in low magnification showing the surface structures of AVIs isolated from the adaxial epidermis of the inner petal region. E. A higher magnification SEM image of the same material as in D. F. Higher magnification SEM image of the boxed region in E. G. Higher magnification SEM image of the boxed region in F.

To understand more about the organization of AVIs in lisianthus petals, we examined them using bright field light microscopy and SEM. At high magnification under bright field, the surfaces of the AVIs in adaxial epidermal cells appeared as a collection of irregular colored deposits and strands that were tangled in the central space of the vacuole (Fig. 3A). The pink area surrounding the AVI appeared to have a higher anthocyanin concentration around membrane-like structures weaving through the area (Fig. 3A). These pink areas became colorless in most cells as the petals further developed. Although the shape of the AVIs was different in outer petal regions than in the inner region, the AVI surfaces were similar (Fig. 3A and 3B). This tangled structure of colored deposits and strands was also apparent for the AVIs isolated from the adaxial epidermis of lisianthus inner petal and placed in water (Fig. 3C). All these examinations of AVIs, both in live cells and as isolated particles, failed to show any evidence of a membrane surrounding the entire AVI.

The surface of isolated lisianthus AVIs was further analyzed using SEM (Fig. 3D–G). Acetone was found unable to dissolve lisianthus AVIs in the preliminary experiments and therefore was used to briefly remove water from the AVI preparations prior to SEM. The physical nature of the AVIs was a loose and porous body consisting of irregular granules, strands and sheets (Fig. 3D and 3E). The morphology of the isolated AVIs under SEM appeared to involve membranous networks that folded as boluses (Fig. 3D and 3E). At higher magnification under SEM, these lisianthus AVIs displayed a structure resembling a coral reef (Fig. 3F and 3G), with rough granular or sandy surfaces.

### Internal structures and formation of AVIs

**Figure 4:**
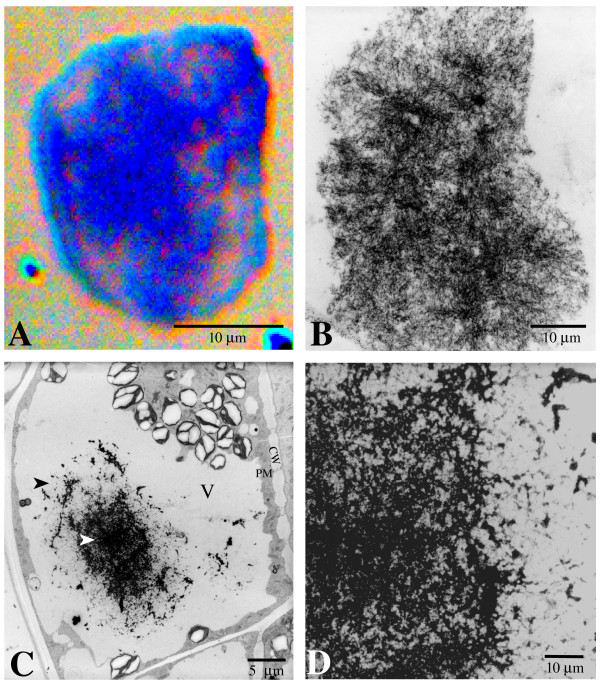
**Micrographs of *in planta *and *in vitro *isolated AVIs of the adaxial cells of the inner petal region of lisianthus flowers**. A. Light microscopy section of an isolated AVI stained with Toluidine Blue, showing the uneven distribution of the internal structure. B. TEM image of an isolated AVI, showing the thread-like structure. C. TEM image of an AVI-containing cell, showing dense inner (white arrowhead) and loose outer thread structures of the AVI in the central vacuole (V). CW, cell wall; PM, plasmodesmata. D. Higher magnification image of the transition part between dense and loose AVI thread structure of an AVI.

To elucidate the internal structures of AVIs, light and TEM examinations were carried out on transverse micro-sections. When the 1 μm sections of the isolated AVIs were stained with Toluidine Blue for light microscopy, blue-colored networks were revealed (Fig. 4A). These networks were unevenly distributed, with some areas being denser than others. The internal ultrastructure observed on the trans-sections was thread-like, with a varied density of electron-dense threads tangled throughout the AVI (Fig. 4B). TEM examinations on the transverse sections again failed to show a membrane encompassing the AVIs (Fig. 4B).

TEM examinations of *in planta *AVIs of adaxial epidermal cells revealed similar structures as the ones identified for isolated AVIs (Fig. 4C and 4D). For the cellular AVIs it was also notable that the outer regions of the thread structure of the AVI residing in the central vacuole in the adaxial cells were more loosely distributed than in the central region of the AVI (Figure 4C). Examination at higher magnification showed that the networks within the cellular AVI seemed to retain more electron-dense materials (Fig. 4D), while the cellular AVI threads resembled those in the isolated AVI. The thickness of the basic AVI threads appeared to be less than 50 nm in both the isolated and the cellular AVIs (Fig. 4B and 4D). No membrane was observed around the intra-vacuolar AVI under TEM (Fig. 4C).

**Figure 5:**
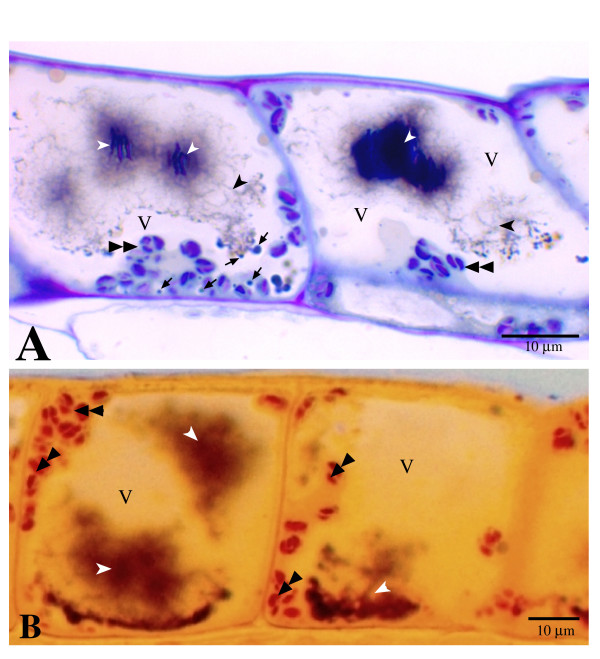
**AVIs observed in transverse section of adaxial epidermal cells of inner petal region under light microscopy**. A. Toluidine Blue stained cells, showing the AVIs have light dense inner structures (white arrowhead) and loose thread network (black arrowhead) around them in the central vacuole (V). Vesicle-like bodies (black arrow) are apparent both in the cytoplasm and in the central vacuole. Chloroplasts, black double arrowhead. B. I_2_-KI stained cells, showing AVI structure (white arrowhead) in the central vacuole (V) but many fewer threads revealed by this staining. Chloroplasts are indicated by double arrowhead.

Staining of the light microscopic sections with Toluidine Blue clearly demonstrated that the central vacuoles in the adaxial epidermal cells of the inner petal region could contain up to several highly condensed 'cores' within an AVI (Fig. 5A, white arrowhead). Surrounding the cores were the loose thread networks (Fig. 5A black arrowhead) that appeared continuous throughout each central vacuole even when they had more than one AVI core. Blue-staining vesicles were clearly observed in the cytoplasm and at the edge of the thread networks in these epidermal cells (Fig. 5A, black arrows). Starch granule-containing chloroplasts were stained pink-purple with Toluidine Blue (Fig. 5A, double black arrowheads). The starch nature in these chloroplasts was further verified using iodine staining (Fig. 5B, double black arrowheads). The thread networks of AVIs were not evident in the unstained and iodine stained sections but the condensed AVI cores were clearly visible (Fig. 1B and 5B).

**Figure 6:**
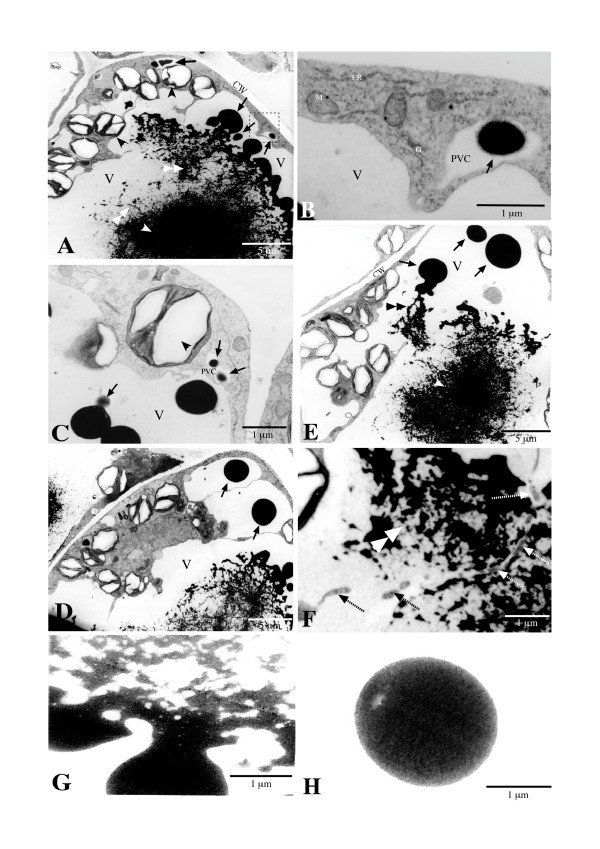
**TEM micrographs of AVIs in the adaxial epidermal cells of the inner petal region of lisianthus flowers**. A. Material being deposited onto a dense AVI part (white arrowhead) from directional rupturing of electron-dense bodies (vesicles, arrow) through a loose thread network zone (double white arrowhead). Smaller electron-dense vesicles are also visible in presumed PVCs in the cytoplasm. B. Close-up image of the boxed region in A, showing a PVC containing an electron-dense vesicle, and the close proximity of the abundant ER. C. Part of an adaxial epidermal cell under high magnification, showing two PVCs (about 250 nm) containing electron-dense bodies (< 200 nm, arrow) in the cytoplasm and a small electron-dense body merging with a large electron-dense body in the central vacuole (V). Starch granule indicated by black arrowhead. D. Part of an adaxial epidermal cell, showing large electron-dense bodies (arrow) in small vacuoles prior to the release to the central vacuole (V). E. TEM image showing electron-dense bodies (arrow) and the rupturing and depositing of its contents (threads, double back arrowhead) onto the dense part (white arrowhead) of an AVI in the central vacuole (V). F. Close-up image of part of an AVI, showing a membranous or thread network and intravacuolar membrane fragments (dashed arrow). G. Close-up image of part of a rupturing electron-dense body. No membrane boundary is apparent. H. Close-up image of an electron-dense body before rupturing. No membrane boundary is apparent.

The formation of AVIs in the adaxial epidermal cells was also investigated by TEM examination of the subcellular structures present. Under TEM, a typical lisianthus adaxial epidermal cell was highly connected with subepidermal cells through numerous plasmodesmata and contained a large central vacuole with a major, irregularly shaped AVI (Fig. 4C). The cytoplasm of these cells had large numbers of endoplasmic reticulum (ER), mitochondria, starch-containing chloroplasts and vesicles (Fig. 6A,6B and 6C). Many of these cytoplasmic vesicles, morphologically resembling the ones revealed under light microscopy (Fig. 5A), contained electron-dense bodies that did not possess clear physical limits, instead displaying a fluffy boundary zone (Fig. 6B and 6C, black arrow).

Although a TEM section is a 'snapshot' of a single time point, there seemed to be a clear transition from the electron-dense bodies in the cytoplasmic vesicles to the AVI in the central vacuole of adaxial epidermal cells (inner petal regions). The accumulation of electron-dense bodies as small as 200 nm (Fig. 6C) was clearly observed in the cytoplasmic vesicles that were morphologically similar to prevacuolar compartments (PVCs) and surrounded by abundant ER (Fig. 6B and 6C). These cytoplasmic vesicles appeared to further develop to various sizes in the PVCs (Fig. 6C and 6D), and the electron-dense bodies in them were released into the central vacuole (Fig. 6D). After release, these electron-dense bodies initially maintained their integrity and trafficked towards the central AVI area and subsequently ruptured so that their contents added to the AVI bulk (Fig. 6D and 6E). The released electron-dense material had a thread-like structure, while the remainder of the ruptured electron-dense body maintained its previous form. These phenomena indicated that these electron-dense bodies are possibly insoluble.

Intravacuolar membrane fragments (Fig. 6F, dash arrow) were sometimes observed among the AVI networks in places. However, even at higher magnification, when the edges of the electron-dense bodies were clearly shown, no evidence of a membrane envelope for the electron-dense body was observed (Fig. 6G and 6H).

## Discussion

Previous studies of AVIs in petals of lisianthus noted the occurrence of thread-like bodies in the outer region of the petal, and larger irregularly shaped bodies in the inner region [20]. From more detailed examination in this study, we can determine three forms of AVIs, which can co-exist in three different types of epidermal cells in the same petal: vesicle-like forms in the abaxial epidermal cells of the inner petal region (Fig. 1A,1D and 1E), irregular forms in the adaxial epidermal cells of the inner petal region (Fig. 1A,1B and 1C) and a rod-like form in the adaxial epidermal cells of the outer petal region (Fig. 2E). It is probable that the three AVI forms reflect differences in the associated vacuolar contents of the different cells, for example the anthocyanin type or amount. The inner region of lisianthus flowers is known to have a different anthocyanin profile to the outer region [20], but it is not known whether the anthocyanins vary between the abaxial and adaxial epidermis. It is clear that flowers have sophisticated mechanisms for controlling the amount and type of pigment produced in specific regions of the petal, to allow complex floral pigmentation patterns to be formed [23]. There are no obvious environmental signals associated with cell location or cell type that would influence the type of AVI that occurs. Light is the main signal that affects AVI formation in maize cell cultures [24], probably through promoting the fusion of anthocyanin-containing vesicles into AVI-like structures that contained the spread of anthocyanins from the inclusions into the vacuolar sap. However, light incidence is likely to be similar for the inner and outer region epidermal cells in lisianthus flowers under glasshouse conditions.

The vesicle, thread and large irregular forms of AVIs observed may represent three successive steps in AVI formation, perhaps linked to the rate and/or species of anthocyanin biosynthesis in the particular cell, with the large irregular-shaped AVI being the final form. The high levels of "unbound" anthocyanins observed in vacuoles containing the vesicle-like or rod-like AVI forms may indicate that the formation of AVIs in these cells has not progressed to the same extent as in the adaxial epidermal cells of the inner petal region. This is further supported by the observations that, firstly, the "unbound anthocyanins" in the outer petal region can become completely associated with larger AVIs in some cells (Fig 3B), and secondly, that the abaxial epidermal cells had the ability to form insoluble AVI-like granules (Fig. 2C). That the large AVIs in inner region adaxial cells are insoluble structures is supported by a number of observations. Firstly, they can be isolated as stable particles. Secondly, protoplasts prepared from cells containing these AVIs do not form spherical cells but have an irregular shape that reflects the presence of the AVIs within the cells (Fig. 2A). The ability of the protoplasts from the adaxial epidermal cells of the inner petal to maintain their original cellular shapes is unlikely to be due to the incomplete removal of the cell walls but more likely to be due to the presence of the AVI. The protoplasts released from the abaxial epidermal cells always tended to be spherical, but in the same digestion solution the protoplasts derived from the corresponding adaxial epidermal cells always tended to keep their original shapes.

It is not known whether AVIs have a physiological role, and consequently, it is not known whether the different forms of AVIs observed have distinct physiological functions. It has been suggested that AVIs may enable the storage of high concentrations of anthocyanins [20], and have a darkening effect on flower color in lisianthus and roses [20,28]. Whether the impact on flower color has arisen through an evolutionary trend to new colors is not known, but it seems probable that the formation of the different AVIs could be a consequence of changes in the biosynthesis and/or intracellular transport of anthocyanins. The throat of lisianthus does have a much darker color than the outer region. The presence of chloroplasts in the adaxial epidermal cells of the inner petal region but not the outer petal region may also contribute to the darker color of this region. Anthocyanins are thought to be present in complex structures in the vacuole that enable strong color formation, involving processes such as self-association, intra- or inter supramolecular copigmentation [25]. Whether these states can still be formed when the anthocyanins are associated with an AVI is not known, but it is possible they may be generated immediately following biosynthesis and before transport to the AVI. These self- and inter-molecular interactions may continue to draw together the components of an AVI to form final structures.

Different forms of AVIs have been reported in different species. For instance, AVIs in the cell cultures derived from sweet potato tubers [22] or grapevines [26] have a vesicle-like morphology similar to that observed in some lisianthus cells. However, electron microscopy examination [22] showed that the sweet potato AVIs in the vacuoles had neither membrane boundary nor internal structures but appeared as strongly osmiophilic globules [22]. AVIs in red-cabbage leaves [27], some cells of lisianthus, carnation [20], rose [28] and apple skins [29] have more compact forms, either regular or irregular in shape, and do not resemble vesicles. Apart from the presence of anthocyanins, and the VP24 protein identified as associated with sweet potato AVIs [30-32], the materials present in AVIs are unknown. As with the sweet potato AVIs, neither the lisianthus (this work and [20]) nor apple [29] AVIs display a membrane envelope. Although there is no single surrounding membrane, we did find evidence for membrane fragments associated with AVIs in lisianthus petals. Both SEM and light microscopy observations of isolated and *in planta *AVIs showed a rough and grainy surface comprised of strand-, granule- and sheet-like structures, suggestive of a membranous origin, and the fine thread network structures observed by TEM also suggest the presence of intravacuolar membranous materials. Although acetone was used during SEM sample preparation, AVIs remained physically intact. This suggests either that the membranous structures observed in the AVIs are not lipid-based, or that the anthocyanins are able to protect these structures from corrupting by acetone.

The results obtained in the current study clearly suggest that, in lisianthus petals, the ultimate AVIs in the central vacuoles are derived from the aggregation of anthocyanins into insoluble structures that are similar to membranous networks in appearance (Fig 4 and 5). The aggregation process seems to have started from some kind of 'seed' structure (Fig. 5A), perhaps composed of membrane material, and then the surrounding anthocyanic networks continue to be deposited to these 'seed' structures (Fig. 4, 5 and 6). A clear traffic route of anthocyanic vesicle-like bodies from the cytoplasm to the central vacuole observed in this study also demonstrates that mass transport of anthocyanins is a major means of anthocyanin sequestration into the central vacuole.

Based on the observations of lisianthus petal cells, a hypothesis can be presented for formation of AVIs in this species. As the anthocyanins are being formed on the ER they may be simultaneously transported into PVCs in the cytoplasm and accumulate as electron-dense vesicle-like bodies. Certainly these colored, electron-dense bodies present in the cytoplasm, as observed with the light and TEM microscopes respectively, indicate that the cytoplasm is not only the site of anthocyanin biosynthesis but also a site where anthocyanins can accumulate to a certain level. Material from the ER may be co-transported with the anthocyanins into these transport bodies, perhaps including membrane fragments. That the vesicle-like bodies do not contain a free solution of anthocyanins is suggested by the images of them rupturing and releasing their contents onto the AVI. The PVCs gradually enlarge and subsequently merge with the central vacuole to release the anthocyanic, electron-dense bodies into the central vacuole. As the material is released from the electron-dense bodies into the main vacuole environment, the network of insoluble anthocyanic thread-like material is then formed and aggregated to give a stable AVI particle. This may occur if the solubility of the material is different between internal vesicle environment and vacuolar environment. If co-pigmentation or self-stacking of anthocyanins is occurring on the growing AVI then this may further promote insolubility.

Vesicles directly derived from ER have been reported to be involved in the mass transport of proteins into protein storage vacuoles in soybean and rice [33,34]. Intracellular trafficking of ER-derived vesicles to the central vacuole has also been suggested for yellow fluorescent phytochemicals in transgenic maize BMS cell suspensions [4], including the formation of AVI-like structures [17]. The accumulation of pre-vacuolar vesicles in the *tds4 *mutant of Arabidopsis [35,36] also suggests an involvement of vesicle transport in proanthocyanin biosynthesis. Further evidence for the formation of vesicles associated with the biosynthesis of anthocyanins on the ER comes from studies of the localisation of the biosynthetic enzymes. UDP-glucose:flavonol 2'- and 5'-*O*-glucosyltransferases in leaves of *Chrysosplenium americanus *[37], and chalcone synthase and chalcone isomerase in Arabidopsis roots [38] have been localized not only on the ER but also in small vesicles, which may be derived from the ER. In the epidermal cells of lisianthus petals, the prevacuoles were often observed in close proximity to abundant ER, although whether this is a casual or coincidental occurrence is not known.

Morphological evidence of intracellular flavonoid transport and sequestration into the central vacuole is required to reconcile with evidence derived from molecular research [6]. Based on the cellular and sub-cellular evidence in the current study, it is tempting to assume that GSTs [9-12,39] and transporters [13,40] mainly work at the stage when anthocyanins are being packed into prevacuoles in the cytoplasm. Losses of anthocyanin-related GSTs or transporters may disrupt the packing process in the cytoplasm. Further studies using combined approaches of molecular and cell biology will help gain more insight into this process.

## Conclusion

AVIs can take three different forms in the epidermal cells of lisianthus, dependent on the positions of the epidermal cells on a petal. Although these different forms are relatively stable, they do seem to have the ability to progress towards the ultimate morphology of an irregular form. The present study has clearly demonstrated that a mechanism involving mass transport of anthocyanins from the cytoplasm to the central vacuole exists in lisianthus petals. Cellular and subcellular evidence suggests that the anthocyanins may be first packed into PVCs in close proximity to the sites of anthocyanin biosynthesis. These prevacuolar vesicles, along with the contained anthocyaninic bodies, further develop and ultimately merge with the central vacuole to deliver anthocyanins into the vacuole, where various forms of AVIs develop. Physically, AVIs in lisianthus are aggregates of anthocyanin-containing membrane-like and thread networks. Further investigation is currently being undertaken to look into chemical nature of these anthocyanic networks in the AVI.

## Methods

### Plant material

The lisianthus used in this study was a deep purple-flowered variety Wakamurasaki (WMS, Mioshi Seed Company, Japan). All plants were grown in pots at 18–24°C throughout the year with no supplemental lighting in a standard glasshouse in Palmerston North, New Zealand. During the experiments, fully open and healthy flowers were harvested, petals were detached and then thoroughly washed at least three times with 1 liter of tap water containing three drops of Tween-20 prior to a final wash with MilliQ water.

### Protoplast Generation

The washed petals were chopped up using an onion chopper on a plastic board until the petals became slurry. Small amount of phosphate buffer (0.1 M, pH 7.0) containing 10 mM EDTA was added during the chopping in order for the chopped petals to form slurry. The petal slurry was added to a macerating solution [41] containing 1% (w/v) cellulase Onozuka R-10 (Yakult Honsha Co., Higashi-Shinbashi, Minatoku, Tokyo) and 0.05% (w/v) Pectolyase Y23. The mix was incubated on a rocker overnight at room temperature to release AVI-containing protoplasts.

### AVI isolation

The method of isolating AVIs from flower petals was based on that of Markham and colleagues [20] with some modifications. The mix of AVI-containing protoplasts was vortexed, filtered through 50-μm cheesecloth and washed in 0.1 M phosphate buffer plus 0.3 M NaCI (wash solution) prior to centrifugation at 100 g for 5 min to collect an AVI-containing pellet. The pellet was washed in combination with vortexes twice more with the wash solution to remove debris and increase free AVIs. The final pellet was suspended in a minimal volume of wash solution.

The suspended pellet was transferred into a new tube containing 80% (v/v) Percoll (AMRAD-Pharmacia Biotech, Auckland, New Zealand) and mixed with vortexing. The pellet containing free AVIs was harvested from the bottom of the centrifuge tube after centrifugation at 10,000 g for 10 min. The pellet was washed twice in wash solution and then stored at -80°C until use.

### Scanning Electron Microscopy

Isolated AVIs were quickly washed twice in 0.1 M phosphate buffer (pH 7.0), once in 100% acetone, and then briefly air-dried at room temperature. The conductive surfaces of the AVIs during the SEM were achieved through gold sputtering, using a BAL-TEC SCD 050 coater. AVIs were then observed and photographed with a Cambridge 250 Mk III scanning electron microscope (SEM).

### Transmission Electron Microscopy

Isolated AVIs, small pieces of carnation petals and lisianthus inner petals were fixed in 0.1 M phosphate buffer (pH 7.2) containing 3% (v/v) glutaraldehyde and 2% (v/v) formaldehyde. After three washes with the phosphate buffer, the samples were transferred to 1% OsO_4 _(w/v) for secondary fixation. Dehydration used a graded acetone series (25%, 50%, 75%, 95% and 2× 100%). The samples were then embedded in Procure 812 epoxy resin at 60°C for 48 h. Ultra-thin sections were prepared using a diamond knife and a Leica Ultracut R Ultramicrotome. Nickel grid-mounted sections were double-stained using saturated uranyl acetate in 50% (v/v) ethanol, followed by lead citrate. Observation and photography used a Philips 201C transmission electron microscope.

### Light Microscopy

Transverse sections (1 to 3 μm thick) of isolated AVIs and petals were prepared in the same way as described for the transmission electron microscope. Prior to light microscopy observation, the sections were stained with 0.05% (w/v) Toluidine Blue in 0.1 M phosphate buffer (pH 7.2), KI/I_2 _solution (containing 2.0% (w/v) KI and 1.0% (w/v) I_2_) or unstained to take advantage of the anthocyanin color as markers. All light microscopy of petal peels, protoplasts, isolated AVIs, sectioned AVIs and petals used an Olympus BH-2 microscope in either bright field or epifluorescent mode. All samples were mounted on glass slides and covered with glass cover slides for observation. Photographs were taken using Leica DFC Cameras operated by Leica FireCam software on a Macintosh computer.

## Authors' contributions

H.Z carried out most of the experimental work and wrote the initial manuscript draft. L.W. contributed to the experimental work, in particular the AVI isolations. R.B. prepared the EM sections. S.D. participated in the investigation on AVI morphology. K.D. contributed to the initiation of the project and was involved in design and supervision of the research and the writing of the manuscript.

## References

[B1] Schwinn KE, Davies KM, Davies KM (2004). Flavonoids.. Plant pigments and their manipulation.

[B2] Davies KM, Davies KM (2004). An introduction to plant pigments in biology and commerce. Plant pigments and their manipulation.

[B3] Quattrocchio F, Wing JF, van der Woude K, Mol JN, Koes R (1998). Analysis of bHLH and MYB domain proteins: species-specific regulatory differences are caused by divergent evolution of target anthocyanin genes. Plant J.

[B4] Grotewold E, Sainz MB, Tagliani L, Hernandez JM, Bowen B, Chandler VL (2000). Identification of the residues in the Myb domain of maize C1 that specify the interaction with the bHLH cofactor R. Proc Natl Acad Sci U S A.

[B5] Davies KM, Schwinn KE (2003). Transcriptional regulation of secondary metabolism. Functional Plant Biology.

[B6] Grotewold E (2006). The genetics and biochemistry of floral pigments. Annu Rev Plant Biol.

[B7] Kitamura S, Grotewold E (2006). Transport of flavonoids. The Science of Flavonoids.

[B8] Marrs KA, Alfenito MR, Lloyd AM, Walbot V (1995). A glutathione S-transferase involved in vacuolar transfer encoded by the maize gene Bronze-2. Nature.

[B9] Alfenito MR, Souer E, Goodman CD, Buell R, Mol J, Koes R, Walbot V (1998). Functional complementation of anthocyanin sequestration in the vacuole by widely divergent glutathione S-transferases. Plant Cell.

[B10] Larsen ES, Alfenito MR, Briggs WR, Walbot V (2003). A carnation anthocyanin mutant is complemented by the glutathione *S*-transferases encoded by maize *Bz2* and petunia *An9*. Plant Cell Rep.

[B11] Kitamura S, Shikazono N, Tanaka A (2004). TRANSPARENT TESTA 19 is involved in the accumulation of both anthocyanins and proanthocyanidins in Arabidopsis. Plant J.

[B12] Mueller LA, Goodman CD, Silady RA, Walbot V (2000). AN9, a petunia glutathione S-transferase required for anthocyanin sequestration, is a flavonoid-binding protein. Plant Physiol.

[B13] Debeaujon I, Peeters AJ, Leon-Kloosterziel KM, Koornneef M (2001). The TRANSPARENT TESTA12 gene of Arabidopsis encodes a multidrug secondary transporter-like protein required for flavonoid sequestration in vacuoles of the seed coat endothelium. Plant Cell.

[B14] Mathews H, Clendennen SK, Caldwell CG, Liu XL, Connors K, Matheis N, Schuster DK, Menasco DJ, Wagoner W, Lightner J, Wagner DR (2003). Activation tagging in tomato identifies a transcriptional regulator of anthocyanin biosynthesis, modification, and transport. Plant Cell.

[B15] Goodman CD, Casati P, Walbot V (2004). A multidrug resistance-associated protein involved in anthocyanin transport in Zea mays. Plant Cell.

[B16] Grotewold E, Chamberlin M, Snook M, Siame B, Butler L, Swenson J, Maddock S, Clair GS, Bowen B (1998). Engineering secondary metabolism in maize cells by ectopic expression of transcription factors. Plant Cell.

[B17] Lin Y, Irani NG, Grotewold E (2003). Sub-cellular trafficking of phytochemicals explored using auto-fluorescent compounds in maize cells. BMC Plant Biol.

[B18] Abrahams S, Lee E, Walker AR, Tanner GJ, Larkin PJ, Ashton AR (2003). The Arabidopsis TDS4 gene encodes leucoanthocyanidin dioxygenase (LDOX) and is essential for proanthocyanidin synthesis and vacuole development. Plant J.

[B19] Pecket RC, Small CJ (1980). Occurrence, location and development of anthocyanoplasts.. Phytochemistry.

[B20] Markham KR, Gould KS, Winefield CS, Mitchell KA, Bloor SJ, Boase MR (2000). Anthocyanic vacuolar inclusions--their nature and significance in flower colouration. Phytochemistry.

[B21] Nozue M, Yasuda H (1985). Occurrence of anthocynoplasts in cell suspension culture of sweet potato. Plant Cell Reports.

[B22] Nozue M, Kubo H, Nishimura M, Katou A, Hattori C, Usuda N, Nagata T, Yasuda H (1993). Characterization of Intravacuolar Pigmented Structures in Anthocyanin-Containing Cells of Sweet-Potato Suspension-Cultures. Plant and Cell Physiology.

[B23] Schwinn K, Venail J, Shang YJ, Mackay S, Alm V, Butelli E, Oyama R, Bailey P, Davies K, Martin C (2006). A small family of MYB-regulatory genes controls floral pigmentation intensity and patterning in the genus Antirrhinum. Plant Cell.

[B24] Irani NG, Grotewold E (2005). Light-induced morphological alteration in anthocyanin-accumulating vacuoles of maize cells. BMC Plant Biol.

[B25] Ellestad GA (2006). Structure and chiroptical properties of supramolecular flower pigments. Chirality.

[B26] Conn S, Zhang W, Franco C (2003). Anthocyanic vacuolar inclusions (AVIs) selectively bind acylated anthocyanins in Vitis vinifera L. (grapevine) suspension culture. Biotechnol Lett.

[B27] Small CR, Pecket RC (1982). The ultrastructure of anthocyanoplasts in red-cabbage. Planta.

[B28] Gonnet JF (2003). Origin of the color of Cv. rhapsody in blue rose and some other so-called "blue" roses. J Agric Food Chem.

[B29] Bae RN, K-W K (2006). Anatomical observations of anthocyanin rich cells in apple skins. HortScience.

[B30] Nozue M, Kubo H, Nishimura M, Yasuda H (1995). Detection and Characterization of a Vacuolar Protein (Vp24) in Anthocyanin-Producing Cells of Sweet-Potato in Suspension-Culture. Plant and Cell Physiology.

[B31] Nozue M, Yamada K, Nakamura T, Kubo H, Kondo M, Nishimura M (1997). Expression of a vacuolar protein (VP24) in anthocyanin-producing cells of sweet potato in suspension culture. Plant Physiol.

[B32] Xu WX, Moriya K, Yamada K, Nishimura M, Shioiri H, Kojima M, Nozue M (2000). Detection and characterization of a 36-kDa peptide in C-terminal region of a 24-kDa vacuolar protein (VP24) precursor in anthocyanin-producing sweet potato cells in suspension culture. Plant Science.

[B33] Mori T, Maruyama N, Nishizawa K, Higasa T, Yagasaki K, Ishimoto M, Utsumi S (2004). The composition of newly synthesized proteins in the endoplasmic reticulum determines the transport pathways of soybean seed storage proteins. Plant Journal.

[B34] Takahashi H, Saito Y, Kitagawa T, Morita S, Masumura T, Tanaka K (2005). A novel vesicle derived directly from endoplasmic reticulum is involved in the transport of vacuolar storage proteins in rice endosperm. Plant Cell Physiol.

[B35] Abrahams S, Tanner GJ, Larkin PJ, Ashton AR (2002). Identification and biochemical characterization of mutants in the proanthocyanidin pathway in Arabidopsis. Plant Physiology.

[B36] Abrahams S, Lee E, Walker AR, Tanner GJ, Larkin PJ, Ashton AR (2003). The 
Arabidopsis TDS4 gene encodes leucoanthocyanidin dioxygenase (LDOX) 
and is essential for proanthocyanidin synthesis and vacuole development. Plant Journal.

[B37] Latchinian-Sadek L, Ibrahim RK (1991). Flavonol ring B-specific O-glucosyltransferases: purification, production of polyclonal antibodies, and immunolocalization. Arch Biochem Biophys.

[B38] Saslowsky D, Winkel-Shirley B (2001). Localization of flavonoid enzymes in Arabidopsis roots. Plant J.

[B39] Pairoba CF, Walbot V (2003). Post-transcriptional regulation of expression of the Bronze2 gene of Zea mays L. Plant Mol Biol.

[B40] Coleman JOD, BlakeKalff MMA, Davies TGE (1997). Detoxification of xenobiotics by plants: Chemical modification and vacuolar compartmentation. Trends in Plant Science.

[B41] Morgan ER (1998). Callus production from protoplasts of Cyclamen persicum.. Plant Cell Tissue and Organ Culture.

